# Aspirin overcomes cisplatin resistance in lung cancer by inhibiting cancer cell stemness

**DOI:** 10.1111/1759-7714.13619

**Published:** 2020-09-29

**Authors:** Maoyuan Zhao, Ting Wang, Zhouguang Hui

**Affiliations:** ^1^ Department of Radiation Oncology National Cancer Center/National Clinical Research Center for Cancer/Cancer Hospital, Chinese Academy of Medical Sciences & Peking Union Medical College Beijing China; ^2^ Department of VIP Medical Services National Cancer Center/National Clinical Research Center for Cancer/Cancer Hospital, Chinese Academy of Medical Sciences & Peking Union Medical College Beijing China; ^3^ State Key Laboratory of Molecular Oncology National Cancer Center/National Clinical Research Center for Cancer/Cancer Hospital, Chinese Academy of Medical Sciences & Peking Union Medical College Beijing China

**Keywords:** Aspirin, cisplatin, lung cancer, stemness

## Abstract

**Background:**

Lung cancer is the leading cause of cancer death and is commonly treated by cisplatin. Although cisplatin treatment may initially be successful, its effectiveness usually reduces significantly in disease‐recurrent patients. Aspirin, a nonselective COX inhibitor, has been shown to help reverse the status of cisplatin sensitivity in recurrent human ovarian cancer cells. This study aimed to explore the effect of aspirin on cisplatin resistance through the perspective of cancer cell stemness.

**Methods:**

We used clustering analysis to predict the H460 cisplatin resistance from the GSE21656 dataset. The increased lung cancer cell stemness may contribute to enhanced tolerance. In this study, we used aspirin, a nonselective COX inhibitor, with cisplatin for several hours in cells and days in vivo, and studied the inhibition against human cisplatin‐resistant H460 cells. H460 cisplatin‐sensitive and H460 cisplatin‐resistant cells were treated with 16 μM aspirin or/and 0.3 μg/mL cisplatin for 72 hours.

**Results:**

H460 cisplatin‐resistant cells showed stronger resistance, stemness, and invasiveness than H460 cisplatin‐sensitive, and cisplatin significantly reduced the survival of cisplatin‐sensitive cells, while cisplatin with aspirin dramatically reduced the surviving fractions of cisplatin‐resistant cells.

**Conclusions:**

This study revealed that stemness is a latent inhibitor of the resistance of lung cancer cisplatin‐resistant cells and might be effectively inhibited by aspirin.

## Graphical abstract

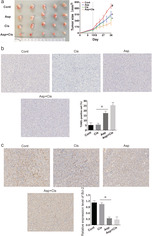

Our research revealed that stemness is a latent inhibitor of the resistance of lung cancer cisplatin‐resistant cells and might be effectively inhibited by aspirin

## Introduction

Lung cancer is the leading cause of cancer death with a less than 20% five‐year survival rate.^1^ Resistance to clinical integrated treatment is reported as one major reason for treatment failure.^2^ The standard treatment of lung cancer is plain‐based combination chemotherapy.^3^ Cisplatin occupies a leading position in platinum anticancer drugs in clinical chemotherapy and integrated treatment of lung cancer.^3^ Therefore, exploring the causes and solutions to cisplatin resistance is crucial for improving treatment outcome of lung cancer.

Despite the lack of a clear underlying mechanism for lung cancer cisplatin resistance,^4^ it is widely accepted that cancer stem cell hypothesis could be one potential mechanism that causes treatment failure and leads to recurrence.^5^ Several studies have shown that stem cell‐related mechanisms could influence the resistance, including the apoptotic mechanism of cell damage, enhanced DNA repair mechanism, upregulation of multidrug resistance proteins, and extramembrane transport proteins.^5^ Among them, the most important is ATP binding cassette (ABC) transporter family including ATP binding cassette subfamily G subfamily member 2 (ABCG2), which regulates and controls the flux across the plasma membrane of chemotherapeutic agents.^6^ ABC transporter‐related genes are highly expressed in hematopoietic stem cells while hardly expressed in terminally differentiated cells.^7^ When they are highly expressed in cancer stem cells, ABC transporter‐related genes pump out small structural unrelated molecules, including cytotoxic chemotherapeutic drugs.^8^ Lung cancer stem cells can express high levels of ABC transporters thus decreasing intracellular drug concentration.^8^ In addition, CD133 is an important stem cell factor. Previous studies have reported that cisplatin elevated CD133 expression in lung cancer postoperative patients,^9^ and CD133‐positive lung cancer patients have a shorter progression‐free survival than CD133‐negative patients.^9^

It has been reported that there is a close correspondence between stemness enhancement of lung cancer cells and the tumor microenvironment (TME) that includes cancer cells, immune cells, etc^10^ The rapid growth of tumors leads to insufficient oxygen supply in the microenvironment, which results in the upregulation of hypoxia‐inducible factor‐1α (HIF‐1α). HIF‐1α activates the COX‐2 signaling axis and is crucial in cancer cell stemness.^11^ A new study found that aspirin blocked the formation of metastatic intravascular niches by inhibiting COX‐1.^12^ Aspirin, a nonselective COX inhibitor, has been illustrated to decrease the risk of lung cancer in epidemiological and clinical studies.^13^, ^14^ Aspirin has also been found to help reverse the status of cisplatin sensitivity in recurrent human ovarian cancer cells.^15^ Thus, we hypothesized that aspirin may alleviate the upregulation of stemness‐related proteins, which might be beneficial to overcome the increase of drug resistance‐related proteins.

This study observed the influences of aspirin in lung cancer cisplatin‐resistant cells using cisplatin‐resistant tumor‐bearing node mice. We evaluated the activities of regulatory factors to predict the H460 cisplatin resistance from the GSE21656 dataset. We hypothesized that aspirin overcame cisplatin resistance by inhibiting lung cancer stemness, and that aspirin might have implications in clinical integrated treatment methods.

## Methods

### Cell lines, culture, shRNA, plasmid

We obtained the human lung cancer cell line H460 cisplatin‐sensitive (H460S) and H460 cisplatin‐resistant (H460R) cells from the National experimental cell resource collection of Chinese Academy of Medical Sciences/Peking Union Medical College (CRC‐PUMC, Beijing, China). The culture environment has been previously described elsewhere.^16^ We purchased cisplatin from Hansoh Pharmaceutical Group Co Ltd (Lianyungang, Jiangsu, China). The recombinant plasmids of CD133 and CD44 were constructed with open reading frames of CD133 and CD44 digested with *HindIII* and *BamHI* and subcloned into pcDNA3.1 (+) plasmids for overexpression, and the CD133 and CD44 short hairpin (shRNA) were contained in liposomes for the knockdown. The empty pcDNA3.1 (+) plasmids were used as negative controls and the vectors already contained scrambled control shRNA sequences.

### In vivo model

NOD/BALB/c mice (female, four‐week‐old; Beijing HFK Bioscience Co., Ltd., Beijing, China) were housed in constant laboratory conditions with a 12 hour light/dark cycle. We subcutaneously injected 5 × 10^5^ H460R cells in 100 μL PBS into the right back of the mice. We randomly assigned the mice with a tumor (0.5–1.0 cm wide, and 0.5–1.0 cm long) into four groups: (i) 100 μL normal saline; (ii) aspirin (50 mg/kg/24 hours); (iii) cisplatin (30 mg/kg/72 hours); and (iv) cisplatin with aspirin. Each group consisted of five mice. We dissolved cisplatin in normal saline by intraperitoneal injection three times a week for 21 days, and aspirin in ultrapure water with 4% ethanol as a cosolvent using the same method once a day for 21 days. The tumor volume (major axis) × (minor axis) was monitored every 2–3 days. The mice were sacrificed 21 days after treatment.^16^

### Cell proliferation (MTT assay)

We seeded 10^4^ cells per well into 96‐well plates, which were cultured for 24, 48, and 72 hours after adding MTT solution to the cells for four hours. They were then analyzed at 560 nm using a microplate reader (Benchmark Electronics, Angleton, TX, USA).

### Flow cytometric analysis of apoptosis

H460R cells were treated and measured as previously described,^16^ double‐stained using fluorescein isothiocyanate (APC)‐conjugated Annexin‐V and propidium iodide (PI) (BD Biosciences, San Jose, CA, USA). PE‐CD133, PE‐CD44, FITC‐CD24 were obtained from eBioscience (Vienna, Austria).

### Western blot analysis

H460R cells were harvested and total protein was quantified using Micro BCA protein assay kit (Pierce, Rockford, IL, USA). We separated total protein (30 μg), incubated the primary antibodies (1:1000) and the corresponding secondary antibodies (1:10000; Cell Signaling Technology, Danvers, MA, USA), and measured the signal as previously described.^17^ Caspase‐3, Bax, PTEN, ABCG2, SMC1, PUMA, CD44, Oct‐4, Sox‐2, HIF‐1α, TGF‐β1, TGF‐βR2, SMAD4, and β‐actin were obtained from Abcam Science Company (Cambridge, UK).

### Terminal deoxynucleotidyl transferase‐mediated dUTP nick end‐labeling (TUNEL) assay

Tumor sections 5 μm in thickness were obtained using microtome and the sections were deparaffinized in xylene and rehydrated in a graded concentration of ethanol. We subsequently incubated the sections with Proteinase K and 30% H_2_O_2_ to increase tissue permeability and diminish any endogenous peroxidase activity. We then incubated the sections with complete labeling reaction buffer and antibody solution. The colorimetric reaction was visualized using 3,3‐diaminobenzidine (DAB). We had three samples for each mouse analyzed over five visual fields for each sample.

### Immunohistochemical analysis

The tumors from NOD BALB/c mice were removed, fixed in 10% formalin and embedded in paraffin. We cut and mounted the sections, and performed immunocytochemical analysis using the recommended method to examine the expressions of HIF‐1α, ABCG2, ALDH1, BCL‐2, and TGF‐β1 (Cell Signaling Technology, Danvers, MA, USA). Five random areas of interest were examined in each section and those identified by computer‐generated field identification.

### Microarray data information

NCBI‐GEO is a free public database of microarray/gene profile, and we obtained the gene expression profile of GSE21656 in H460 cisplatin‐sensitive cells and H460 cisplatin‐negative cells on GPL6244 Platforms (HuGene‐1_0‐st) Affymetrix Human Gene 1.0 ST Array (transcript [gene] version) which included three H460 cells and three resistant cells.

### Enriched pathway analysis

Gene set enrichment analysis (GSEA) was conducted using the software GSEA v4.0.3 (www.broadinstitute.org/software/gsea). The H460 cisplatin‐resistant cell gene expression level was annotated as high or low phenotype, and YAMASHITA_LIVER_CANCER_STEM_CELL_DN was utilized. We deleted CYP3A4, C1R, PIK3C2G, APOA5 and added CD24, CD44, ABCG2 due to differences between lung cancer stem cell markers^17^ and liver cancer stem cell markers.^18^ All other parameters were set as default.

### Statistical analysis

We used analysis of variation (ANOVA) and *t*‐test for statistical analysis and the data are presented as mean ± standard deviation (SD). *P* < 0.05 was considered statistically significant. All statistical analyses were performed using SAS 9.2.

## Results

### H460 cisplatin‐resistant (H460R) cells showed stronger stemness than H460 cisplatin‐sensitive (H460S)

**Figure 1:**
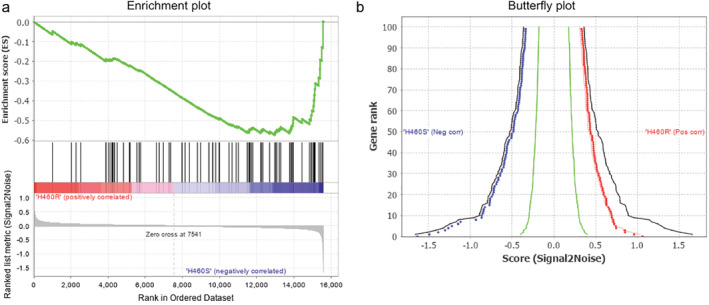
Bioinformatic analysis of H460R and H460S. (**a**) The GSEA results show the correlation of stem related gene sets (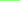) Enrichment profile, (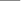) Hits, (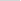) Ranking metric scores. (**b**) Shows the relationship between the first (the right curve) and the last 100 (the left curve) genes and the signal‐to‐noise ratio (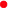) Observed pos, (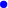) Observed neg, (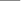) Permuted pos 1%, (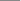) Permuted neg 1%, (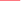) Permuted pos 5%, (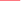) Permuted neg 1%, (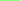) Permuted pos 50%, (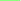) Permuted neg 50%.

To investigate the possible mechanism of cisplatin resistance, we performed GSEA between H460R and H460S cells expression datasets of GSE21656. Stemness showed the most significantly enriched plot in Fig 1(a), and the curves in Fig 1(b) on both sides were not scattered, indicating the results in Fig 1(a) were reliable.

### H460R cells showed stronger resistance, stemness, and invasiveness, and aspirin relieved enhancement in vitro

**Figure 2:**
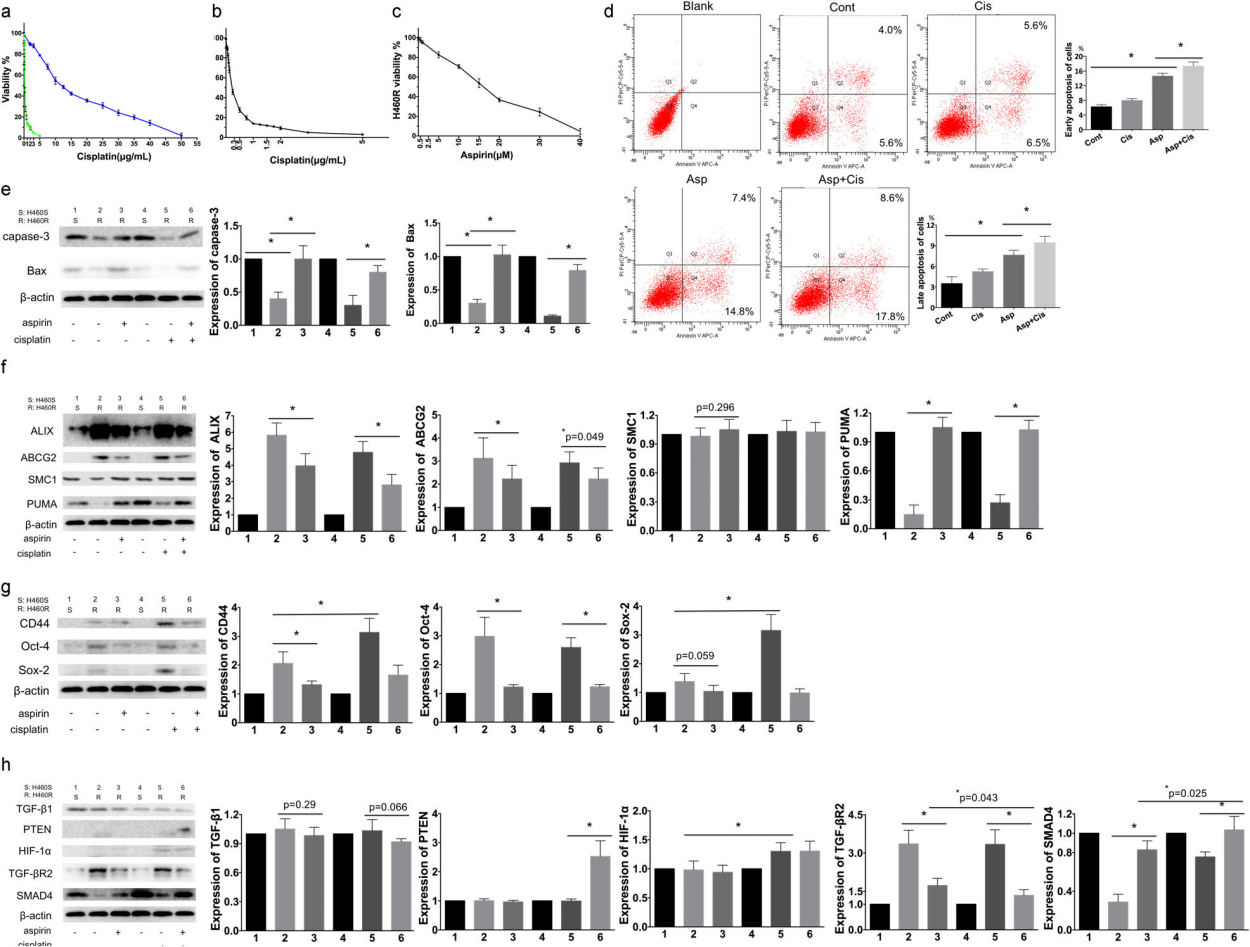
Aspirin relieve the enhancement resistance, stemness and invasiveness of H460R cells in vitro. H460S and H460R with 0–5 μg/mL (0–17 μmol/L) or 0–50 μg/mL(0–170 μmol/L) of cisplatin, respectively, for 72 hours. Cell viability was evaluated by MTT assay. (**a**) H460R and H460S were assayed for cisplatin sensitivity (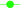) H460S, (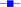) H460R. (**b**) H460S was assayed for cisplatin sensitivity. Average and standard deviations (SD) of three independent experiments are presented. (**c**) Aspirin sensitivity of H460R after a 72 hours treatment. Cell viability was evaluated by MTT assay, 50% ± 3% survival at 16 μM. (**d**) H460S cells were stained with Annexin V‐APC and propidium iodide following treatment with or without 16 μM aspirin or 0.3 μg/mL cisplatin for 72 hours. Apoptosis was determined by flow cytometry. Cont, control. Regulation of proteins affecting (**e**) apoptosis, (**f**) drug resistance, (**g**) stemness; and (**h**) invasion and metastasis. Protein levels were detected by western blotting. β‐actin was used as the loading control. Numbers 1 and 4 were H460S cells treated without aspirin or cisplatin, and number 2, 3, 5, 6 were H460R cells. Number 2 was untreated. Numbers 3, 5, 6 were treated with aspirin, cisplatin, and aspirin with cisplatin, respectively. In the same strip, the density of 1 and 4 might not appear exactly the same because it was impossible for all parts of the strips to be applied to the same concentration of developer at the same time. We calculated the average value and SD of repeating the experiments for statistically significant times, so the density of 1 and 4 on the strip graph is slightly different, which does not affect our multiple experimental results. Values are expressed as means ± SD, *n* = 5, *P < 0.05.

We observed the integrity of the cisplatin‐sensitive and cisplatin‐resistant lung cancer cell line H460, H460S IC50 and H460R IC50 dose of cisplatin, and the effect of cisplatin on the colony‐forming ability of the cells (Fig 2a). H460S IC50 dose was regarded as the subsequent dose of cisplatin for later experiments. More subtle dose ranges and cell viability are presented in Fig 2b to illustrate the H460S IC50 dose clearly. The marked reduction was in H460S cells after a low dose cisplatin treatment (eg, 9% ± 2% survival at 2 μg/mL) and complete inhibition was at a dose of 5 μg/mL. The IC50 dose of cisplatin for the H460S cells was 0.3 μg/mL. On the other hand, H460R cells showed cisplatin resistance. The observed IC50 dose of cisplatin for the H460R cells, 16 μg/mL, was approximately 43 times higher than that for H460S cells. These results indicated H460R cell resistance to cisplatin treatment.

MTT assay was performed to evaluate the effect of different aspirin concentrations on the proliferation of H460R cells. Aspirin led to an inhibition of cell viability, 50% ± 3% survival at 16 μM (Fig 2c). Flow cytometry was used to assess apoptosis of H460R cells following treatment with 0.3 μg/mL cisplatin and/or 16 μM aspirin (Fig 2d). Early apoptosis was PI‐/APC+ and late apoptosis was PI+/APC+. We analyzed the expression of apoptosis‐related proteins (Fig 2e), drug resistance‐related proteins (Fig 2f), stemness related proteins (Fig 2g), cancer invasion and metastasis‐related proteins (Fig 2h). After 72 hours treatment, western blotting revealed decreased levels of Bax and caspase‐3 in H460R, and increased levels of Bax and caspase‐3 following treatment with aspirin or aspirin mixed with cisplatin, while single cisplatin showed no change. As for drug resistance‐related proteins, western blotting showed increased levels of ABCG2 in H460R, single aspirin and aspirin with cisplatin relieved the increased level, but single cisplatin did not. PUMA showed an opposite trend of protein expression with ABCG2 in H460R and single aspirin and aspirin with cisplatin played a similar role. However, there was no statistically significant difference in SMC1 after 72 hours treatment with H460S and H460R. CD44, Oct‐4, Sox‐2 all reduced in H460R. Aspirin reduced the increase in CD44 and Oct‐4; however, there was no statistical significance in reducing the expression of Sox‐2 in H460R cells. Single cisplatin increased CD44 and Sox‐2 expression. Aspirin with or without cisplatin showed no statistically significant difference in expression of stemness related proteins in H460R cells. PTEN showed a meaningful increase when aspirin was combined with cisplatin. HIF‐1α upregulation was notable in combined treatment and aspirin only, and there was no difference in the two groups. Western blotting results showed decreased levels of SMAD4 and increased levels of TGF‐βR2 in H460R compared with H460S, and the combination showed the most conspicuous change.

### Cisplatin with aspirin inhibited growth of H460R tumors in vivo

**Figure 3:**
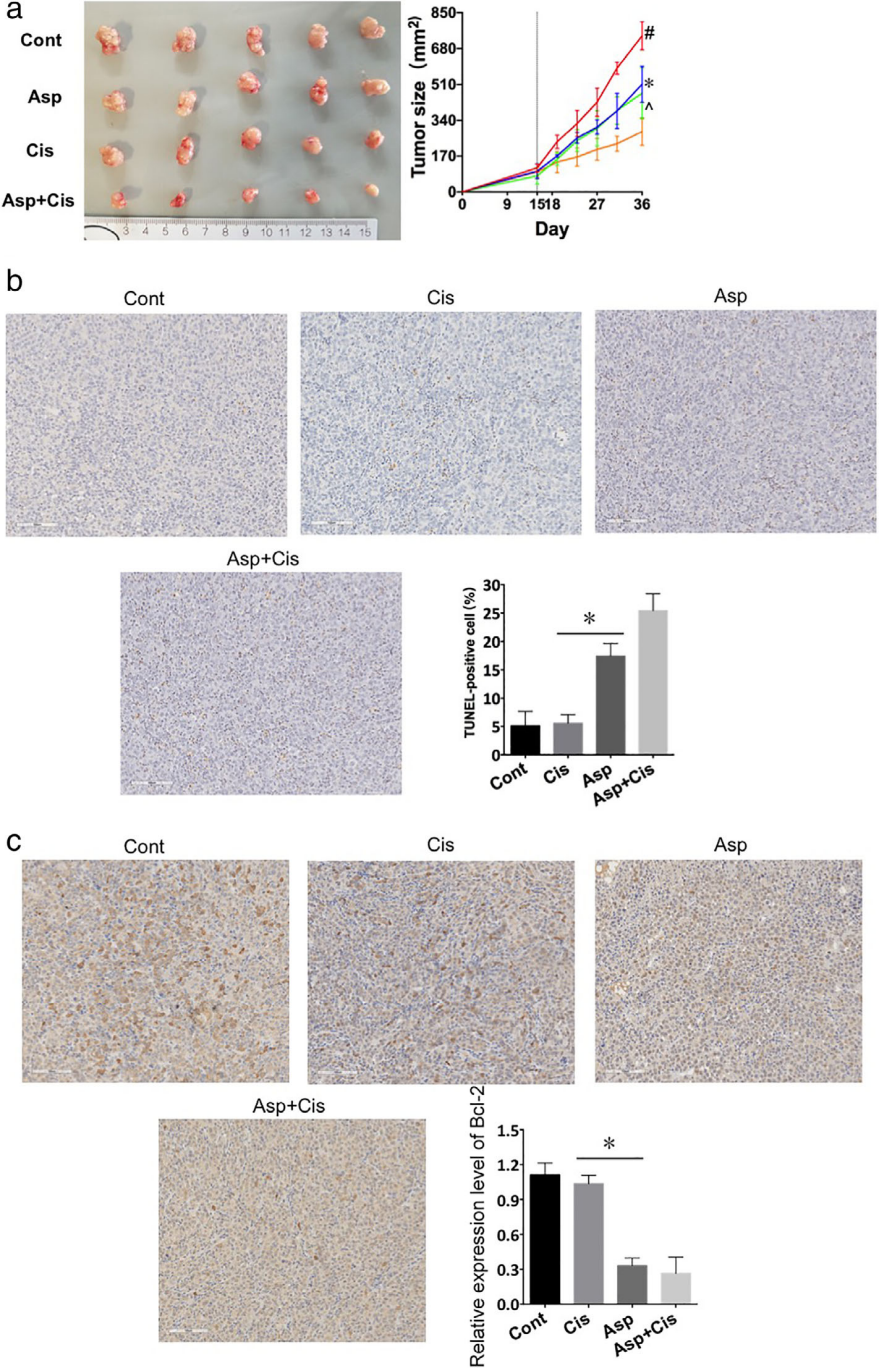
Aspirin with cisplatin inhibited the growth of H460 tumors. Asp, aspirin; Cis, cisplatin; Cont, control. (**a**) The administration of aspirin with cisplatin significantly inhibited H460R tumor growth. Tumor size in the Asp + Cis group was the smallest while that in control animals was the largest. Tumor sizes in the Asp and Cis group were smaller than that in the Cont group. Tumor sizes after treatment of BALB/c nude female mice with 50 mg/kg aspirin and 30 mg/kg cisplatin. The right panel shows changes in tumor size in each group, and the left panel shows representative images of the tumors. Values are expressed as means ± SD, *n* = 5; #*P* < 0.05 versus the Asp, Cis, Asp + Cis groups at the same time point; *P < 0.05 versus Asp + Met groups at the same time point. ^*P* < 0.05 versus Asp + Met groups at the same time point (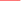) Cont, (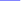) Asp, (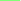) Cis, (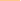) Asp+Cis. (**b**) Apoptosis assayed with TUNEL staining (magnification, x200). The Cont group showed the least apoptosis while the Asp + Cis group showed the most. Values are expressed as means ± SD, *n* = 3, *P < 0.05. (**c**) BCL‐2 expression in H460R transplantation tumors with Asp (ES) or Cisplatin (Cis) treatment for 21 days. The positive rate was highest in the Asp + Cis group. *P < 0.05 compared with the Cis group. Values are expressed as means ± SD, *n* = 3.

The combination of aspirin and cisplatin led to an effective inhibition of tumor growth in vivo, whereas aspirin or cisplatin alone resulted in mild inhibition (Fig 3a). The group treated with the combination agents had the smallest tumor size while the control group had the largest. We did not find a significant difference in tumor sizes between the aspirin and cisplatin groups. We used TUNEL assay and BCl‐2 immunohistochemical analysis to observe apoptosis in subcutaneously transplanted tumors in mice (Fig 3b). In addition, we observed the Bcl‐2 expression level in different treatment groups by immunohistochemical analysis (Fig. 3c). It revealed aspirin induced apoptosis in the H460R tumor, and aspirin with cisplatin showed the strongest promotion in apoptosis. We concluded that the combination contributed to the largest increase in apoptosis, which meant that aspirin overcame cisplatin resistance and the combination played a synergistic role in inhibiting cisplatin‐resistant tumor growth in vivo.

### Cisplatin with aspirin improved hypoxia and inhibited the stemness of H460R tumors in vivo

**Figure 4:**
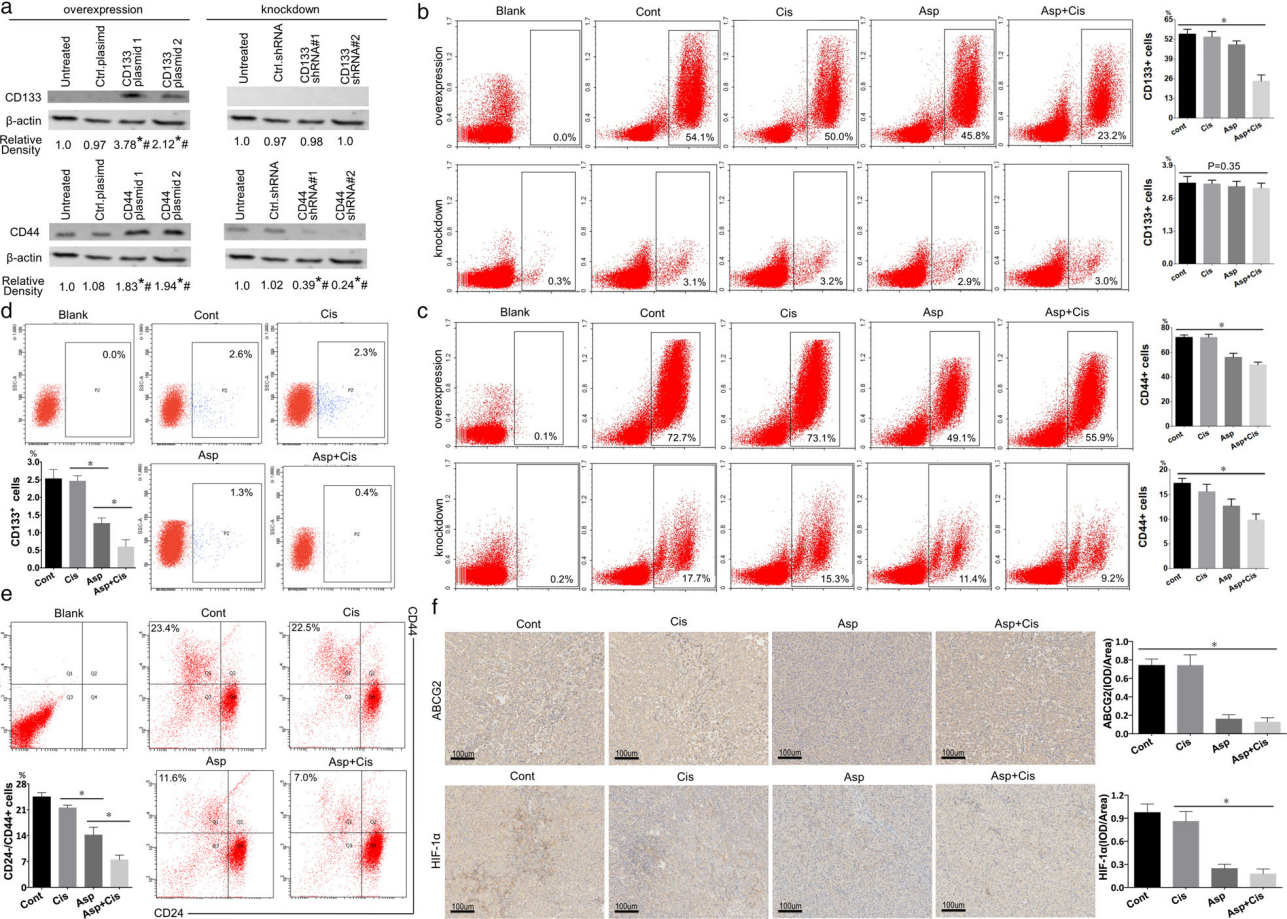
(**a**) Flow cytometry showed that CD133 positive cell amount was higher in the Cont and Cis groups, and *P < 0.05 compared with the Asp and Asp + Cis groups. Values are expressed as means ± SD, *n* = 3. (**b**) Flow cytometry showed that CD24‐/CD44+ cells amount was lowest in the Asp + Cis group and *P < 0.05 compared with Asp, Cis, and Cont groups. Values are expressed as means ± SD, *n* = 3. (**c**) ABCG2 and HIF‐1α expression in H460R transplantation tumors with aspirin (Asp) or cisplatin (Cis) treatment for 21 days. The positive rate was highest in the Asp + Cis group. *P < 0.05 compared with the Cont and Cis groups. Values are expressed as means ± SD, *n* = 3.

To test the effect of aspirin and cisplatin on CD133 and CD44, four sets of different shRNAs (CD133 and CD44 knockdown shRNA#1 and #2) and four sets of different plasmids (CD133 and CD44 overexpression plasmid 1 and 2) specific targeting CD133 and CD44 were stably transfected into highly invasive H460R cells, and their CD133 and CD44 transfectants were shown in Fig 4a. Then, we injected H460R cells which overexpressed or knockdown CD133 or CD44 into the mice, CD133 and CD44 overexpression and CD44 knockdown H460R cells all showed a decrease in aspirin and aspirin with cisplatin group, but the CD133 knockdown did not show obvious significant change in a different group on account that the CD133 expression in all groups of H460R cell was very low (Fig 4b,c). On the other hand, CD133+ cells (Fig 4d) and CD24−/CD44+ cells (Fig 4e) amount in untreated H460R cells were higher in the control and cisplatin groups and lower in the aspirin and aspirin with cisplatin groups, signifying that aspirin overcame cisplatin resistance by inhibiting stemness. The result showed a decreasing trend of ABCG2 and HIF‐1α in the aspirin and aspirin with cisplatin groups, which was consistent with the lightening of H460R tumor stemness, invasion and metastasis (Fig 4f). The results implied that aspirin and cisplatin showed a synergistic effect on lung cancer cisplatin resistance by inhibiting cancer cell stemness.

## Discussion

Resistance rates to cisplatin treatment and carboplatin treatment are as high as 63% and 68% in NSCLC patients.^19^ Therapy‐resistant cancer stem cells might have contributed to the failure of platin‐based conventional therapies.^5^ Hence, distinct cancer stem cell inhibitors could be potential anti‐tumor agents for effective treatment of cancer. In this study, we have unveiled the inimitable role of aspirin in sensitizing stemness of lung cancer cells.

The effect of combining aspirin and cisplatin on the growth of H460 lung cancer cisplatin resistant cells was evaluated in vitro and in vivo. The results of our study revealed a close relationship between cisplatin resistance and strengthened lung cancer cell stemness and a link between the inhibition of lung cancer cell stemness and development of tumor apoptosis in vitro and vivo. The combination of aspirin and cisplatin had a significant inhibitory effect on in vivo tumor growth through regulating important apoptosis‐related proteins, such as Bax and caspase‐3, which led to consequent cell death. The combination was effective at decreasing drug resistance, stemness, and hypoxia in TME. Most of our results are in line with recent literature.^20^, ^21^

In cisplatin‐resistant cell lines, the increased activation of ALDH1 is related to the expression of tumor stem cell markers.^22^ Lung cancer cells expressing CD44 are enriched for stem‐like properties, and CD44‐positive cells show a higher level of expression for pluripotency markers (Oct‐4, Sox‐2) and increased resistance to cisplatin.^24^ CD24‐/CD44+ cancer cells show more significant stemness and resistance than CD24+/CD44+ cancer cells, and there is inherent resistance to radiotherapy.^24^, ^25^ A previous study also reported that patients with the dual expression of CD133 and ABCG2 are at higher risk for tumor recurrence.^26^ PUMA serves as a member of Bcl‐2 family.^27^ It has been shown that PUMA and caspase‐3 activity is abnormally reduced, suggesting that DNA damage‐induced apoptosis caused by cisplatin is suppressed.^28^

The functional relevance of SMAD4 is further supported by emergence of invasive and metastatic in mice tumors and SMAD4 is related to the state of PTEN.^29^ SMAD4 downregulates HIF‐1α expression to improve hypoxia whose inactivation promotes malignancy and drug resistance,^31^ and TGF‐βR2 has a similar effect.^32^ This study demonstrated that aspirin and aspirin with cisplatin suppressed H460R cells. We tested apoptosis proteins such as Mcl‐1, Bax, PUMA and capase‐3 to follow the same trend as that reported in previous studies,^27^, ^28^ and determined that aspirin and aspirin with cisplatin could relieve the apoptosis decrease in lung cancer cisplatin resistant cells. In addition, we observed the depression of ALDH1, Sox‐2 and Oct‐4, CD44 and CD133 with aspirin treatment, accompanied by increased apoptosis and PTEN.

Salicylic acid is aspirin's primary metabolite,^32^ and patients taking low‐dose aspirin excrete salicyluric and *salicylic* acids in their urine.^33^ For in vitro, the concentration of aspirin was 16 μM which had little effect on the pH value of the culture environment. We detected that the pH values were 7.26 in the control group, 7.157 in the aspirin group, 7.213 in the cisplatin group, and 7.137 in aspirin with cisplatin group (Supplementary Fig 1). There was no statistically significant difference between them. Besides, to further confirm the effect of PH value changes from the aspirin on H460R cells, we additionally set the aspirin group, citric acid group, acetic acid group, and vitamin C group, and adjusted their pH value to the same as the aspirin group. After 72 hours, apoptosis was detected by flow cytometry (Supplementary Fig 2). Only aspirin group showed significant early and apoptosis (#, <0.05). For in vivo, the micro‐environment around a tumor had a much lower pH (around 6.8), on account that cancer‐associated fibroblasts drove *lactate production*.^34^, ^35^ For autoregulation and steady‐state regulation in vivo, the effect of normal dose of aspirin on pH of TME is negligible.^36^

Cancer stem cells promote the metastatic and *invasive* ability.^37^ Aspirin reduces invasion of tumors in orthotopic mice xenografts when combines with gemcitabine^38^ and inhibites the formation of metastatic intravascular niches thus decreasing the number of metastatic lung nodules in experimental mice model.^12^ Our results are similar though we found no *distant organ metastasis*, which might be attributable to cell line specificity and that mice were sacrificed before distant metastasis occur, due to the limitation of the longest diameter in animal ethics.

Further studies are needed in the future to address several questions. The study was not able to clearly establish the involvement of stemness in cell signaling and the antiproliferative action of aspirin and cisplatin in lung cancer cells. The mechanism of the inhibition of aspirin with cisplatin may be entirely different from that of cisplatin or aspirin. However, results from the present study using cells in culture and animals are useful for clinical practice.

In summary, this study showed the potential of aspirin to sensitize stemness of H460R cells and overcome cisplatin resistance in lung cancer. This combinatorial strategy could be effective for treatment‐resistant lung carcinomas.

## Disclosure

No conflicts of interest are reported.

## Supporting information


**Figure S1**. The PH value of the four group. Cont: untreated; asp:16 μM aspirin; cis: 0.3 μg/ml cisplatin, asp+cis: 16 μM aspirin with 0.3 μg/ml cisplatin.

**Figure S2**. H460R cells were stained with Annexin V‐APC and propidium iodide following treatment with aspirin group with 16 μM aspirin, citric acid group, acetic acid group, and vitamin C group, and adjusted their pH value to the same as the aspirin group. After 72 hours, the apoptosis was detected by flow cytometry (#, <0.05).
