# Supplementary figures and images for: Aspirin overcomes cisplatin resistance in lung cancer by inhibiting cancer cell stemness
Source: Thorac Cancer. 2020 Sep 29;11(11):3117–25. doi: 10.1111/1759-7714.13619 (PMC7605995; doi:10.1111/1759-7714.13619)

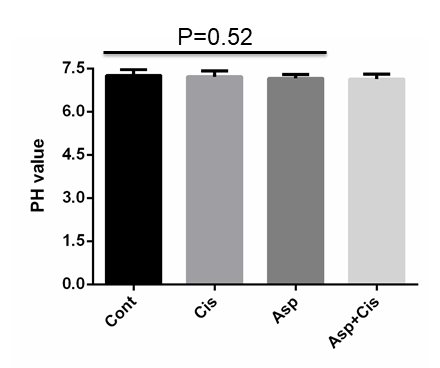

Supplement: Supplementary file 1 — Figure S1. The PH value of the four group. Cont: untreated; asp:16 μM aspirin; cis: 0.3 μg/ml cisplatin, asp+cis: 16 μM aspirin with 0.3 μg/ml cisplatin. [file TCA-11-3117-s001.tif]

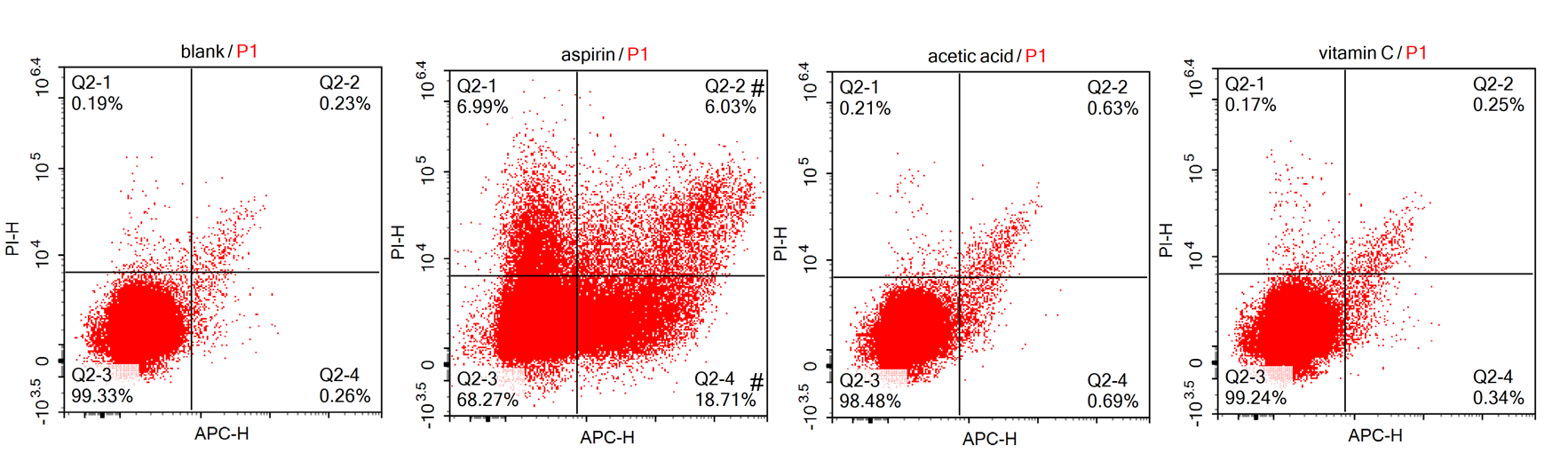

Supplement: Supplementary file 2 — Figure S2. H460R cells were stained with Annexin V‐APC and propidium iodide following treatment with aspirin group with 16 μM aspirin, citric acid group, acetic acid group, and vitamin C group, and adjusted their pH value to the same as the aspirin group. After 72 hours, the apoptosis was detected by flow cytometry (#, <0.05). [file TCA-11-3117-s002.tif]
